# Modulation of proteostasis counteracts oxidative stress and affects DNA base excision repair capacity in ATM-deficient cells

**DOI:** 10.1093/nar/gkx635

**Published:** 2017-07-24

**Authors:** Mattia Poletto, Di Yang, Sally C. Fletcher, Iolanda Vendrell, Roman Fischer, Arnaud J. Legrand, Grigory L. Dianov

**Affiliations:** 1 CRUK & MRC Oxford Institute for Radiation Oncology, University of Oxford, Department of Oncology, Old Road Campus Research Building, Oxford OX37DQ, UK; 2 TDI Mass Spectrometry Laboratory, Target Discovery Institute University of Oxford, Nuffield Department of Medicine, Oxford OX37FZ, UK; 3 Institute of Cytology and Genetics, Russian Academy of Sciences, Lavrentyeva 10, Novosibirsk 630090, Russian Federation

## Abstract

Ataxia telangiectasia (A-T) is a syndrome associated with loss of ATM protein function. Neurodegeneration and cancer predisposition, both hallmarks of A-T, are likely to emerge as a consequence of the persistent oxidative stress and DNA damage observed in this disease. Surprisingly however, despite these severe features, a lack of functional ATM is still compatible with early life, suggesting that adaptation mechanisms contributing to cell survival must be in place. Here we address this gap in our knowledge by analysing the process of human fibroblast adaptation to the lack of ATM. We identify profound rearrangement in cellular proteostasis occurring very early on after loss of ATM in order to counter protein damage originating from oxidative stress. Change in proteostasis, however, is not without repercussions. Modulating protein turnover in ATM-depleted cells also has an adverse effect on the DNA base excision repair pathway, the major DNA repair system that deals with oxidative DNA damage. As a consequence, the burden of unrepaired endogenous DNA lesions intensifies, progressively leading to genomic instability. Our study provides a glimpse at the cellular consequences of loss of ATM and highlights a previously overlooked role for proteostasis in maintaining cell survival in the absence of ATM function.

## INTRODUCTION

Ataxia telangiectasia (A-T) is a rare multisystemic autosomal recessive disorder. The clinical features of the syndrome include progressive neurological impairment, predisposition to cancer and hypersensitivity to ionising radiation (1). A-T is generally linked with mutations in the A-T mutated (ATM) gene, which ultimately lead to the synthesis of a dysfunctional ATM protein (2,3).

ATM is a large serine/threonine kinase belonging to the PI3K-like protein kinase family (4). The protein has been extensively linked with the DNA damage response to DNA strand breaks (5,6) and to reactive oxygen species (ROS) (7). In fact, the presence of widespread oxidative stress constitutes a major feature in A-T and elevated ROS levels have been detected in ATM knock-out mice (8), as well as in lymphocytes from A-T patients (9). ROS are potentially harmful to a number of cellular macromolecules, including DNA and proteins. Oxidative DNA damage is generally dealt with by the DNA base excision repair pathway (BER), which is responsible for the clearance of base lesions and DNA single-strand breaks (SSBs) (10). Importantly, endogenous DNA lesions arise spontaneously at an astounding rate, mainly as a consequence of cellular oxidative metabolism (11), therefore detection and repair of these lesions is absolutely essential to maintain genomic stability. Recent evidence strongly suggests that ATM is a vital sensor for endogenous DNA strand breaks, as its activation has been shown to enforce a cell-cycle delay necessary for DNA repair to occur prior to DNA replication (6,12). Accordingly, impairment of ATM functions affects the G_1_/S checkpoint transition resulting in unrestricted replication of damaged DNA and genomic instability (6,12). While the role of ATM in the context of DNA damage has been thoroughly characterised, much less investigated is the cellular response to ROS-induced protein damage in ATM-deficient cells. Despite the accumulation of ROS and genomic instability, it is clear that a lack of functional ATM is compatible with cell survival, suggesting that adaptation mechanisms must be in place to prevent cell death in the presence of persistent oxidative stress. Nonetheless, the cellular adjustments that promote survival of ATM-deficient cells have been poorly investigated to date.

In this study, we exploit a stable isotope labelling with amino acids in cell culture (SILAC)-based proteomics approach to gain insight into the early adaptation of human fibroblasts to the lack of ATM. Our data confirm that loss of ATM leads to progressive accumulation of ROS and mitochondrial damage, which start very early on upon depletion of ATM. Furthermore, we show that a profound rearrangement of cellular proteostasis takes place in response to ATM depletion and that this is necessary for cells to counter protein damage originating from persistent oxidative stress. Surprisingly, while modulation of proteostasis promotes survival of ATM-depleted cells, this has a considerably negative impact on the BER pathway, whose capacity shows signs of strong impairment. As a consequence, spontaneously generated DNA damage cannot be completely repaired in ATM-depleted fibroblasts, leading to accumulation of genomic instability.

Our study provides insight into cellular adaptation to the loss of ATM, reinforcing the notion that oxidative stress and impaired DNA repair capacity play a major role in the pathology. Moreover, our data highlight a previously overlooked role for proteostasis in maintaining cellular viability in the absence of functional ATM.

## MATERIALS AND METHODS

### Cell culture, chemicals and siRNA transfections

TIG1 and GM03349 normal human fibroblasts, as well as AG03058 A-T fibroblasts were obtained from the Coriell Institute Cell Repository. Cells were grown in DMEM (Life Technologies) supplemented with 15% FBS at 37°C in a humidified atmosphere with 5% CO_2_.

H_2_O_2_ and MMS were from Sigma, MG132 was from Enzo Life Sciences, bortezomib was from Cayman Chemical, and staurosporine was from Merck Millipore. The ATM kinase inhibitors Ku-55933 and Ku-60019 were from Abcam and R&D Systems, respectively.

siRNA transfections were carried out as previously described (13). A detailed list of the siRNAs used can be found in the Supplementary Materials and Methods. Control transfections were carried out using a non-targeting siRNA (Eurogentec, SR-CL000-005). A sequential approach was adopted for simultaneous transfections: ATM was first depleted using 30 nM ATM-targeting siRNA, followed by transfection with the indicated siRNA 24 hours later. The second siRNA was used at a concentration of 5 nM, for a total time of 48 h, allowing us to achieve a moderate depletion for selected BER components.

### Stable isotope labelling with amino acids in cell culture (SILAC) proteomics

SILAC experiments were performed essentially as described in (14) with minor modifications. Briefly, TIG1 cells transfected with a control siRNA were grown in ‘light’ DMEM (R_0_K_0_ – Dundee Cell Products), whereas ATM-depleted cells were labelled in ‘heavy’ DMEM (R_10_K_8_ – Dundee Cell Products). Seventy two hours post transfection cells were counted, pooled and nuclear/cytoplasmic extracts were generated. Samples were precipitated using methanol:chloroform and digested using immobilised trypsin (Pierce-Perbio), desalted using C18 tips (Glygen) and separated by nano-ultraperformance liquid chromatography tandem mass spectrometry (nUPLC-MS/MS) over a 2 hour 1–35% acetonitrile gradient (flow rate was 250 nl/min) coupled to an Orbitrap Fusion Lumos MS (Thermo Fisher Scientific). In brief, full MS scans were acquired using an Orbitrap mass analyser over an *m/z* range of 400–1500 at a resolution of 120k (AGC target of 4e5 ions). MS/MS scanse were acquired in a data-dependent manner with parallel data acquisition (duty cycle of 3 s, dynamic exclusion 30 s). MS/MS spectra were acquired the ion trap in rapid mode following precursor quadrupole isolation with a window of 1.2 and CID fragmentation at 35% normalized collision energy with a maximum injection time of 250 msec. Analysis of SILAC MS data was carried out using the MaxQuant software (v1.5.5.1) as described in (14). LC–MS/MS data was searched against the Human UniProt database (FASTA file released June 2016) using the MaxQuant Andromeda search engine. SILAC data were firstly ranked by calculating the associated *Z*-score. Top-hit proteins (*Z*-score > 2) were used for a gene ontology (GO) enrichment analysis using the GOrilla tool (*http://cbl-gorilla.cs.technion.ac.il/*). Enriched terms provided a list of candidate pathways that were subsequently integrated with proteins sharing the GO function, process or pathway. Integration was performed by manual selection of hits from the SILAC dataset that showed a similar trend of increased expression.

### Flow cytometry

Determination of intracellular ROS content was carried out by staining with 2′,7′-dichlorodihydrofluorescein diacetate (H2DCFDA) (Sigma). Cells were treated as indicated and harvested by trypsinization before loading with 10 μM H2DCFDA in PBS at 37°C for 30 min. Relative ROS content was expressed as a normalised H2DCFDA/FSC ratio. Mitochondrial membrane potential (ΔΨm) was measured using the JC-1 dye (Thermo Fisher Scientific). Cells were harvested as for ROS determination and loaded with 2 μM JC-1 in PBS at 37°C for 30 min. Relative ΔΨm was expressed as a normalized ratio between the mean green and red fluorescence emission of the JC-1 dye. Apoptosis was measured using the AnnexinV-FITC Apoptosis Detection kit (Abcam) as per manufacturer's instructions.

Samples were acquired using a Becton-Dickinson FACSCalibur™ instrument and data analysis was carried out using either BD CellQuest Pro or FlowJo.

### Western blot

Whole cell extracts for Western blot were prepared as described previously (15). Nuclear and cytoplasmic cell extracts were generated as described in (14). A list of the antibodies used in this study can be found in the Supplementary Materials and Methods. Protein carbonylation was assessed using the Oxidized Protein Western Blot Kit (Abcam) with slight modifications. Briefly, 20 μg of cell extract were denatured in 6% SDS and labeled with the 2,4-dinitrophenylhydrazine solution provided with the kit for 1 min at 25°C. The labelling reaction was halted using the provided Neutralization Solution and proteins were resolved by SDS-PAGE. DNP-labelled proteins were probed using the primary antibody provided with the kit; an IRDye^®^800-labelled secondary antibody was used for detection.

### In vitro assays

*In vitro* BER assays were carried out essentially as described in (16), with minor modifications. Briefly, for AP site incision assays, 50 ng of whole cell extract was incubated in 25 mM Tris–HCl pH 7.4, 1 mM MgCl_2_, 100 mM KCl and 1 mM DTT at 37°C for the indicated time. For nick ligation reactions, 2.5 μg of nuclear extract was incubated in 50 mM Tris–HCl pH 7.5, 10 mM MgCl_2_, 10 mM DTT and 1 mM ATP at 25°C for the indicated time. All the oligonucleotide substrates were used at 50 nM and have been previously described (16). DNA substrates were 5′-labelled with IRDye^®^800 (IDT). Reactions were halted with 96% formamide and 10 mM EDTA and analysed by electrophoresis on a 20% denaturing polyacrylamide gel. The percentage of substrate converted to product was determined by using an Odyssey image analysis system (Li-Cor Biosciences).

Proteasome activity was assessed using a fluorimetric assay essentially as described in (17). Nuclear cell extracts were generated as described (14), in the absence of protease inhibitors and adjusted to equal protein concentration. Chymotrypsin activity was measured by incubating 5 μg of nuclear extract in 100 mM Tris–HCl pH 8.0, 10 mM MgCl_2_, 2 mM ATP in the presence of 50 μM Suc-LLVY-AMC (R&D Systems) at 37°C for 50 min. Proteolytic activity was obtained by measuring fluorescence emission (Ex. 380 nm, Em. 460 nm) using a POLARstar Omega plate reader (BMG Labtech). Proteasome-specific activity was calculated by subtraction of the residual chymotrypsin-like activity in presence of 10 μM MG132 (17).

### Viability assays

Cell viability was assessed using a Trypan blue exclusion assay. Briefly, cells were seeded as technical triplicates, treated with the relevant proteasome inhibitor and harvested by trypsinisation. Before counting, cells were diluted two-fold with 0.4% (w/v) Trypan Blue Stain (Life Technologies) and counted with a Countess^®^ Automated Cell Counter (Thermo Fisher Scientific).

### Protein translation assays

Protein translation was assessed using a modified version of the reporter-based assay described in (18). Briefly, cells were treated as indicated; 48 h after treatment, cells were transfected with a pEGFP-N1 plasmid. Plasmid transfection was carried out as previously described (13). Twenty four hours after transfection cells were harvested by trypsinisation, counted, and EGFP fluorescence from equal amount of cells was assessed using a POLARstar Omega plate reader (BMG Labtech). Cells from the same experiment were subjected to RNA extraction and qPCR in order to measure the mRNA expression of EGFP. Translation efficiency was defined as the normalized ratio between the reporter signal and its transcript expression level. Extraction of total RNA, reverse transcription and qPCR were performed as described before (13). The comparative CT method was applied for quantification of gene expression; either *B2M* or *GAPDH* was used as endogenous control. A list of the primers used in this study is reported in the Supplementary Materials and Methods.

### Comet assays and immunostaining

Alkaline and neutral comet assays were carried out as previously described (6). High-throughput microscopy was carried out as described in (13). Protein aggregation was assessed using the PROTEOSTAT^®^ Aggresome detection kit (Enzo Life Sciences) as per the manufacturer's indications. Mitochondrial integrity was monitored using the MitoTracker^®^ Red CMXRos reagent (Thermo Fisher Scientific) as per manufacturer's recommendations.

### Statistical analyses

Statistical analyses were performed by using the two-tailed Student's t-test using either Microsoft Excel or SPSS (IBM). Sample size and standard deviation are indicated for each experiment.

## RESULTS

### Cells lacking ATM undergo progressive accumulation of oxidative stress

The phenotype of cells lacking functional ATM has been extensively characterized. However, most studies have relied on immortalised A-T cell lines obtained from patients, or stable knock-down models. Although extremely valuable, these cell models have substantial limitations, as ATM-deficient cells are genetically unstable (1,19). Therefore, long-term propagation in cell culture will likely lead to unpredictable variations in the cellular genotype and potentially conceal early adaptive changes induced by the absence of ATM.

In order to understand the early stages defining the adaptation of normal cells to the loss of ATM, we compared human diploid fibroblasts treated with an ATM-targeting siRNA (siATM) with their control counterpart (i.e. treated with a non-targeting siRNA, siCtrl). Three days after transfection with the relevant siRNA, cells were subjected to fractionation into nuclear and cytoplasmic compartments and their proteome was analyzed by a SILAC-based quantitative proteomics approach (Supplementary Figure S1A).

**Figure 1.**
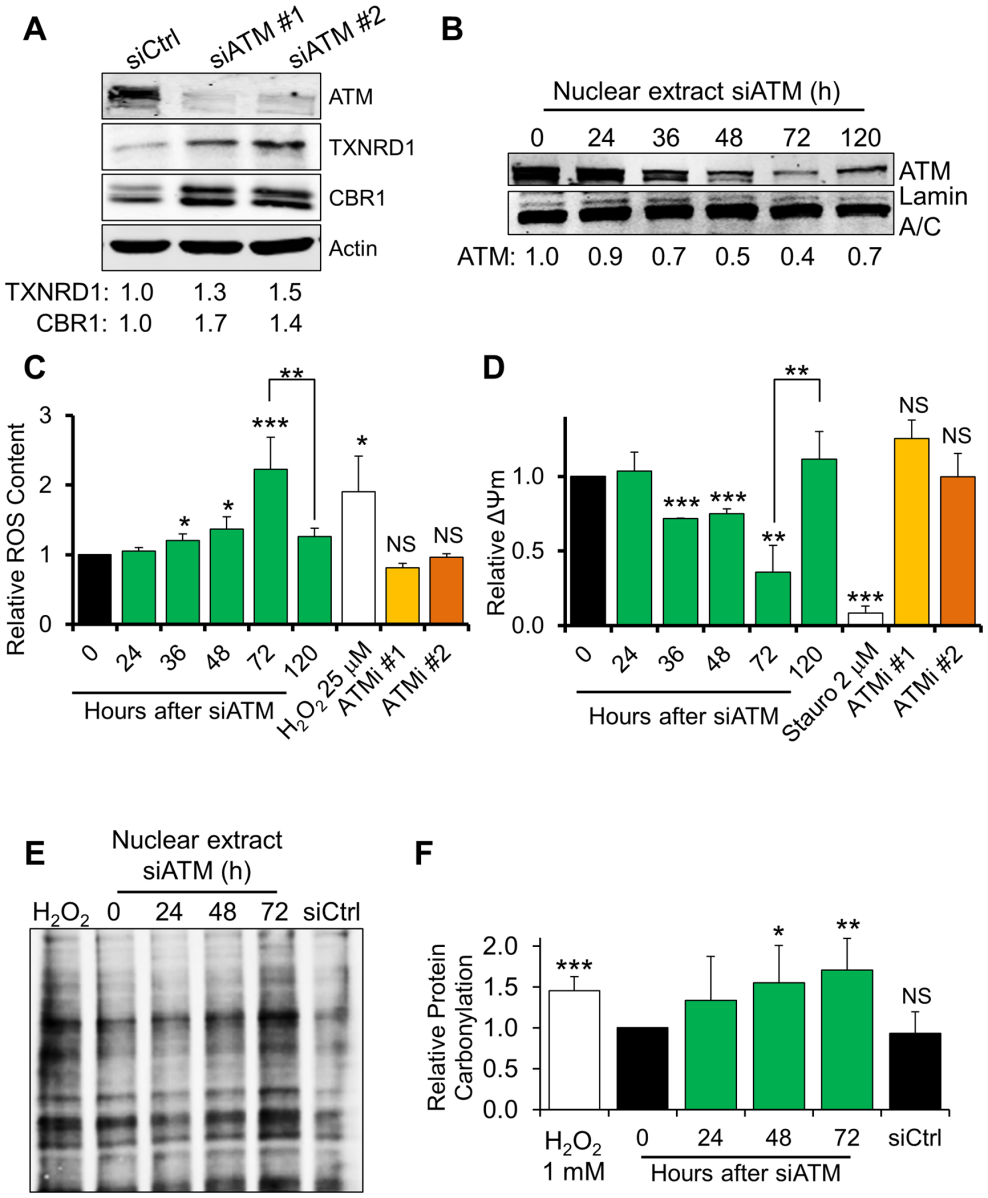
ATM-depleted fibroblasts progressively accumulate ROS and oxidative protein damage. (**A**) Representative Western blot analysis on ATM-depleted fibroblasts showing upregulation of proteins involved in the antioxidant response. TIG1 cells were transfected with either a control siRNA (siCtrl) or an ATM-targeting siRNA (siATM#1, siATM #2). Actin was used as loading control. Densitometric quantification of the indicated proteins is reported at the bottom of the gel (*N* = 2). (**B**) Western blot analysis on a representative time course depletion of ATM. TIG1 fibroblasts were treated as indicated and ATM expression was monitored in nuclear cell extracts. Lamin A/C was used as loading control. (**C**) Quantification of ROS content using flow-cytometry in siATM-treated fibroblasts. TIG1 fibroblasts were treated as indicated. H_2_O_2_ (25 μM, 30 min) was used as a positive control for induction of ROS. ATM inhibitors (Ku-55933 – ATMi #1 and Ku-60019 – ATMi #2, both 10 μM) were provided fresh every 24 h for a total of 72 h (*N* = 3). (**D**) Quantification of mitochondrial membrane potential (ΔΨm) in siATM-treated fibroblasts using flow-cytometry. Cells were treated as in panel C. Staurosporine (2 μM, 2 h) was used as a positive control for mitochondrial membrane depolarisation (*N* = 3). (**E**) Detection of protein carbonylation in nuclear extracts obtained from fibroblasts depleted of ATM for the indicated time. Carbonylated proteins were detected by derivatisation with dinitrophenylhydrazone (DNP) followed by Western blot using an anti-DNP antibody. Equal amounts of cell extract were loaded in each lane. H_2_O_2_ (1 mM, 30 minutes) was used as a positive control for induction of protein carbonylation. (**F**) Quantification of protein carbonylation in ATM-depleted fibroblasts; the histogram displays densitometric data from the analysis reported in panel E (*N* = 6). Results are expressed as mean ± SD from the indicated number (*N*) of independent experiments: *P < 0.05; **P < 0.01; ***P < 0.001; NS: not significant.

Analysis of the proteomics data highlighted a broad upregulation of a number of proteins involved in redox homeostasis (Table 1). Validation of a subset of the SILAC hits confirmed increased expression of carbonyl reductase 1 (CBR1) and thioredoxin reductase 1 (TXNRD1) (Figure 1A). Furthermore, qPCR analyses highlighted that a number genes involved in the antioxidant response, such as CBR1, glutaredoxins (i.e. GLRX, GLRX5), TXNRD1 and peroxiredoxins (i.e. PRDX1, PRDX6), were also upregulated at the transcriptional level (Supplementary Figure S1B). This indicates a broad transcriptional response to transient ATM depletion. Importantly, this was observed using different ATM-targeting siRNAs (Figure 1A and Supplementary Figure S1B).

**Table 1.** Proteins involved in redox homeostasis

UniProt accession number	Description	Gene name	Average fold change upon siATM (nucleus)	Average fold change upon siATM (cytoplasm)
E9PQ63	Carbonyl reductase [NADPH] 1	CBR1	1.60	1.57
P35754	Glutaredoxin-1	GLRX^a^	ND	1.81
Q86SX6	Glutaredoxin-related protein 5, mitochondrial	GLRX5^a^	ND	1.91
P07203	Glutathione peroxidase 1	GPX1	ND	1.13
E9PHN7	Glutathione S-transferase Mu 2	GSTM2	ND	1.47
P21266	Glutathione S-transferase Mu 3	GSTM3	ND	1.43
P78417	Glutathione S-transferase omega-1	GSTO1	1.21	1.21
P09211	Glutathione S-transferase P	GSTP1	1.25	1.25
P09601	Heme oxygenase 1	HMOX1^a^	1.69	ND
B4DLR8	NAD(P)H dehydrogenase [quinone] 1	NQO1	1.49	1.38
Q06830	Peroxiredoxin-1	PRDX1^a^	1.82	1.42
P32119	Peroxiredoxin-2	PRDX2	1.31	1.04
P30041	Peroxiredoxin-6	PRDX6	1.39	1.36
P32322–2	Pyrroline-5-carboxylate reductase 1, mitochondrial	PYCR1^a^	1.24	1.56
Q96C36	Pyrroline-5-carboxylate reductase 2	PYCR2	1.10	1.21
Q9H3N1	Thioredoxin-related transmembrane protein 1	TMX1	ND	1.33
Q9Y320–2	Thioredoxin-related transmembrane protein 2	TMX2	1.14	ND
Q96JJ7	Protein disulfide-isomerase TMX3	TMX3^a^	1.89	1.00
Q86UY0	Thioredoxin domain-containing protein 5	TXNDC5	1.26	1.36
O43396	Thioredoxin-like protein 1	TXNL1	1.49	1.21
E9PIR7	Thioredoxin reductase 1, cytoplasmic	TXNRD1^a^	1.72	1.73

^a^Statistically significant hits from the SILAC analysis (*Z*-score > 2). The table reports the normalised siATM/siCtrl ratio obtained calculating the average of two technical replicates.

ND: not detected in the proteomics analysis.

Activation of the antioxidant response may be triggered by accumulation of ROS; consistently, several studies indicate that ATM impairment is potentially linked with increased oxidative stress (8,9,20). A time course analysis upon siRNA-mediated ATM depletion revealed that the protein levels gradually decreased, with the effect of the siRNA peaking 72 h post-transfection (Figure 1B). Notably, ATM levels started to increase again 120 hours after siRNA transfection (Figure 1B). In agreement with our proteomics data showing elevated levels of antioxidant proteins, flow-cytometry analyses using a ROS-sensitive probe revealed a time-dependent accumulation of ROS in ATM-depleted cells (Figure 1C). Significant ROS accumulation occurred as early as 36 h upon depletion of ATM and ROS levels were mainly restored when the siRNA effect tapered off (Figure 1B and C). ROS increase was also evident in untransformed fibroblasts obtained from an A-T patient (Supplementary Figure S1C). However, upon prolonged (72 h) exposure to specific inhibitors of ATM’s kinase activity (Supplementary Figure 1D), ROS levels did not change significantly (Figure 1C).

Appearance of ROS in ATM-depleted cells was paralleled by disruption of the mitochondrial network (Supplementary Figure S1E) and progressive decline in mitochondrial membrane potential, measured by flow-cytometry using a mitochondrial membrane potential-specific probe (Figure 1D). Again, this phenotype was reverted upon ATM re-expression (Figure 1D). Disruption of the mitochondrial network was also readily observed in fibroblasts from an A-T patient (Supplementary Figure S1F). However, incubation of normal fibroblasts with ATM kinase inhibitors did not affect mitochondrial membrane potential (Figure 1D). These data point to a decline in mitochondrial function in cells lacking ATM, as suggested by earlier studies (21,22). Altogether, these observations demonstrate that a lack of ATM, but not inhibition of its kinase activity, leads to ROS accumulation and loss of mitochondrial organisation and membrane potential.

DNA, lipids and proteins are all affected by ROS, which induce covalent modifications in these cellular components. Protein oxidation can interfere with cellular homeostasis, often leading to protein aggregation, unfolding and loss of activity (23). As ATM-depleted cells showed accumulation of ROS, we assessed whether this led to oxidative modification of cellular proteins. Detection of protein carbonylation in cell extracts obtained from fibroblasts depleted of ATM was carried out by protein derivatisation with dinitrophenylhydrazone (DNP) followed by detection with an anti-DNP antibody, as described in ‘Materials and Methods’. Consistent with the progressive build-up of intracellular ROS, a time-dependent increase in carbonylated proteins was observed in ATM-knockdown cells, especially in the nuclear compartment (Figure 1E and F). Notably, accumulation of oxidised protein species was reverted after ATM restoration (Supplementary Figure S2A). Protein oxidation was readily detected in fibroblasts from an A-T patient (Supplementary Figure S2B), but not after inhibition of the ATM kinase activity in normal fibroblasts (Supplementary Figure S2C); this was entirely consistent with unchanged ROS levels in cells treated with ATM inhibitors (Figure 1C).

These data show that the immediate response to ATM depletion leads to damage to mitochondria, early accumulation of ROS and progressive increase in the intracellular content of oxidised protein.

### ATM-depleted cells counteract ROS by modifying protein turnover

As ATM knock-down fibroblasts showed substantial accumulation of oxidised protein species (Figure 1), we investigated the consequences of this phenomenon. Sustained protein oxidation has been shown to affect protein turnover (23). In line with these observations, our proteomics analysis revealed upregulation of stress-responsive inhibitors of general translation such as EIF2AK2, EIF2AK4, EIF4A2 and ISG15 (18,24,25) in siATM-treated cells (Table 2). This suggests that protein translation might be affected in ATM-depleted cells.

**Table 2.** Proteins involved in the unfolded protein response and translation control

UniProt accession number	Description	Gene name	average fold change upon siATM (nucleus)	Average fold change upon siATM (cytoplasm)
O95817	BAG family molecular chaperone regulator 3	BAG3^a^	ND	1.59
Q16543	Hsp90 co-chaperone Cdc37	CDC37	1.44	1.34
Q9UBS4	DnaJ homolog subfamily B member 11	DNAJB11	1.32	1.37
Q9UDY4	DnaJ homolog subfamily B member 4	DNAJB4	ND	1.27
X6R9L0	DnaJ homolog subfamily C member 3	DNAJC3	1.39	1.27
P19525	Interferon-induced, double-stranded RNA-activated protein kinase	EIF2AK2	1.26	1.20
H0YME5	Eukaryotic translation initiation factor 2-alpha kinase 4	EIF2AK4	1.38	ND
P38919	Eukaryotic initiation factor 4A-II	EIF4A2^a^	2.67	2.74
Q9BS26	Endoplasmic reticulum resident protein 44	ERP44	1.52	1.28
P08107	Heat shock 70 kDa protein 1A	HSPA1A	1.28	1.32
P11021	78 kDa glucose-regulated protein	HSPA5	1.45	1.44
P05161	Ubiquitin-like protein ISG15	ISG15^a^	ND	1.81
K7EJE8	Lon protease homolog, mitochondrial	LONP1	1.41	0.86
M0R208	ATP-dependent Clp protease proteolytic subunit, mitochondrial	CLPP	ND	1.24

^a^Statistically significant hits from the SILAC analysis (*Z*-score > 2). The table reports the normalised siATM/siCtrl ratio obtained calculating the average of two technical replicates.

ND: not detected in the proteomics analysis.

**Figure 2.**
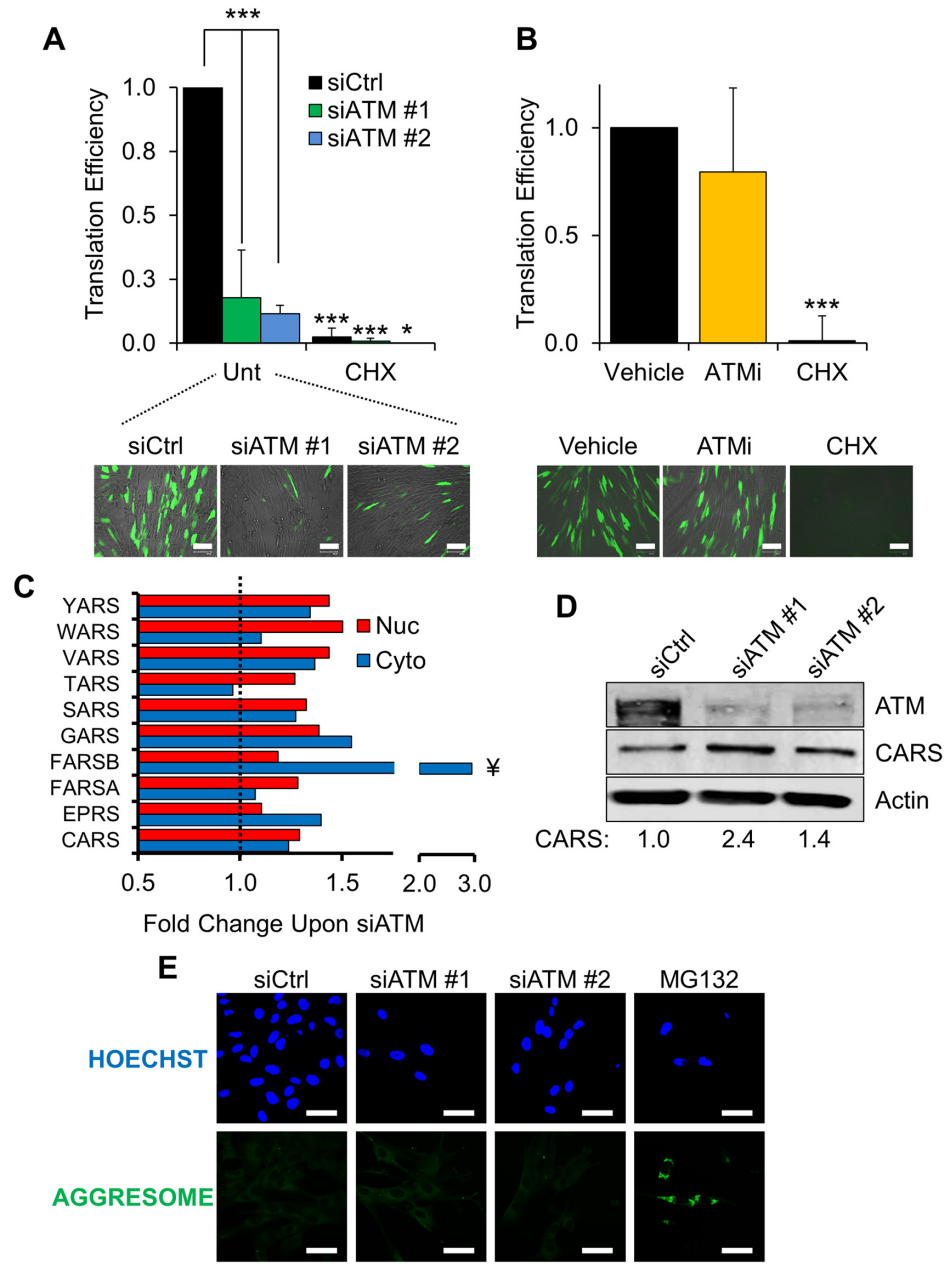
Fibroblasts lacking ATM show changes in protein biosynthesis. (**A**) Protein translation assay. TIG1 fibroblasts were transfected with the indicated siRNA and subsequently with an EGFP reporter, as indicated in ‘Materials and Methods’. Translation efficiency was determined by calculating the ratio between the fluorescence of the reporter (*bottom panel*) and EGFP transcription. ATM-depleted cells show significantly lower translation efficiency than control fibroblasts. Cycloheximide-treated cells (CHX, 25 μg/ml for 24 hours) were used as positive control for translation suppression. Scale bar in the representative micrographs is 100 μm (*N* = 3). (**B**) Translation assay carried out as in panel A. Cells were treated with either vehicle (DMSO) or ATM kinase inhibitor (KU-55933, 10 μM for 72 h, fed fresh every 24 h). Scale bar 100 μm (*N* = 3). (**C**) Proteomics data showing the fold change in aaRSs expression upon ATM depletion. Red and blue bars show the change in protein expression in the nuclear and cytoplasmic fractions, respectively. Data are expressed as the average fold change calculated between two technical replicates. The dashed line represents the normalised expression level in cells transfected with the control siRNA. ¥: statistically significant hits (*Z*-score > 2). (**D**) Validation of the SILAC data using Western blot. The expression level of CARS was measured 72 h after transfection with different ATM-targeting siRNAs. Densitometric quantification of CARS is reported at the bottom (*N* = 2). (**E**) Immunofluorescence analysis on TIG1 fibroblasts treated with the indicated siRNA. Aggresome formation was detected as described in ‘Materials and Methods’. MG132 (5 μM for 16 h) was used as positive control for induction of protein aggregates. Nuclei were stained with Hoechst. Scale bars 50 μm. Results are expressed as mean ± SD from the indicated number (N) of independent experiments: *P < 0.05; ***P < 0.001.

In order to test this hypothesis, we determined the rate of general protein translation using a reporter-based assay. An EGFP-expressing plasmid was transfected into siCtrl- or siATM-treated fibroblasts and the rate of translation was measured as a ratio between the fluorescent signal of the reporter and its transcript levels (18). In line with our SILAC data showing upregulation of inhibitors of general translation, cells depleted of ATM displayed a strong impairment in protein translation ability (Figure 2A). This phenotype, however, was not observed when ATM kinase activity was inhibited (Figure 2B).

In addition to changes in protein translation, analysis of our proteomics data highlighted increased expression of a number of aminoacyl tRNA synthetases (aaRSs), Figure 2C. Notably, most of the upregulated aaRSs are responsible for the esterification of aromatic amino acids (e.g. tyrosine - YARS, tryptophan—WARS, phenylalanine—FARSA and FARSB) and cysteine (i.e. CARS), which are all particularly prone to oxidation (26,27) (Figure 2C). Using Western blot analyses we confirmed upregulation of CARS using two different ATM-targeting siRNAs (Figure 2D). aaRSs normally promote amino acid ligation to transfer RNAs; however, in order to promote translation fidelity, aaRSs can also catalyse hydrolysis of modified amino acids using a mechanism known as ‘editing’ (27). Given our data showing that ATM-depleted fibroblasts upregulate selected aaRSs, we can speculate that increased aaRS expression could either stimulate incorporation of undamaged amino acids into newly synthesised proteins, or prevent incorporation of oxidised amino acids into nascent polypeptides.

Further analysis of our proteomics data revealed that ATM depletion leads to upregulation of molecular chaperones involved in the unfolded protein response such as heat shock proteins, ERP44 (28) and DnaJ proteins (Table 2). Surprisingly, however, ATM-depleted fibroblasts did not show any accumulation of aggresomes, as assessed using immunofluorescence (Figure 2E). This evidence suggests that adjustments to protein translation were sufficient to prevent the unfolding and aggregation of oxidised proteins in the absence of functional ATM.

### ATM-deficient cells have increased nuclear proteasome activity

**Figure 3.**
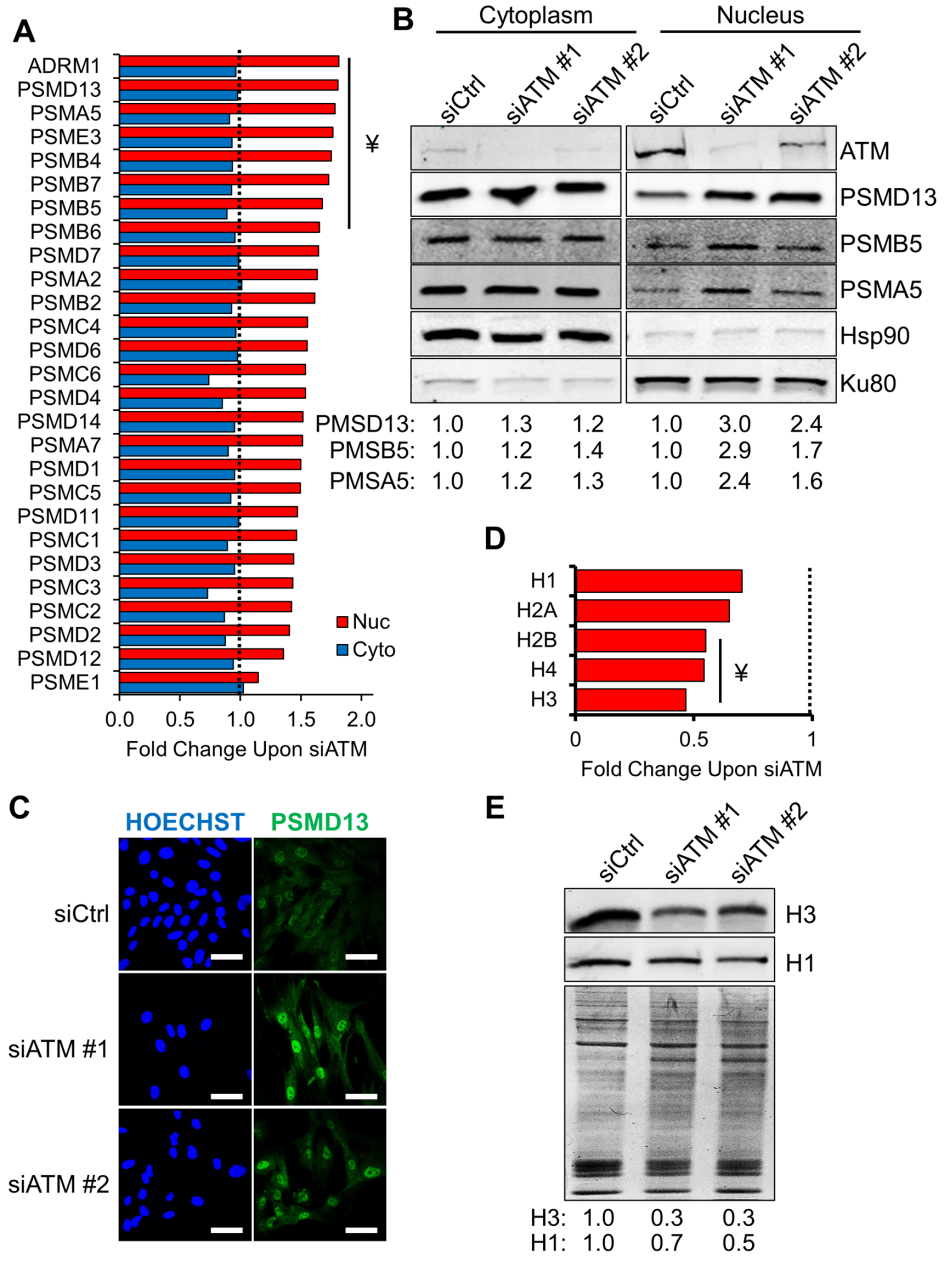
Accumulation of nuclear proteasome and degradation of histones in ATM-depleted fibroblasts. (**A**) Proteomics data showing the fold change of proteasome subunit levels in fibroblasts depleted of ATM. Red and blue bars show the change in protein expression in the nuclear and cytoplasmic compartments, respectively. Data shown are expressed as the average fold change calculated between two technical replicates. The dashed line represents the normalised protein expression level in cell transfected with the control siRNA. ¥: statistically significant hits (*Z*-score > 2). (**B**) Validation of the SILAC data using Western blot. Levels of the indicated proteasome subunits were analysed in cytoplasmic and nuclear fractions after ATM depletion for 72 h using the indicated siRNA. Hsp90 and Ku80 were used as loading controls for the cytoplasmic and nuclear compartment, respectively. Densitometric quantification of the indicated proteins is reported at the bottom (*N* = 2). (**C**) Validation of the SILAC data by immunofluorescence. Localisation and expression levels of PSMD13 were analysed upon ATM depletion for 72 h using the indicated siRNA. Scale bars 50 μm. (**D**) Proteomics data showing the decrease in histone protein levels upon ATM depletion. The histogram shows the change in protein expression in the nuclear fraction. Data shown are expressed as the average fold change calculated between two technical replicates. The dashed line represents the normalised histone expression level in cells transfected with the control siRNA. ¥: statistically significant hits (*Z*-score < 2). (**E**) Representative Western blot analysis validating the results presented in panel A and showing a decreased amount of histones upon ATM depletion. TIG1 fibroblasts were treated with the indicated siRNA and acid-extracted protein fractions were analysed 72 h post transfection. A Coomassie-stained gel is used to show equal loading. Densitometric quantification of the indicated proteins is reported at the bottom (*N* = 2). Results are expressed as mean from the indicated number (*N*) of independent experiments.

Turnover of oxidised proteins requires the concerted action of multiple pathways: on the one hand, proteostasis is maintained through the modulation of protein translation efficiency. On the other hand, proteasomes and lysosomes have been shown to promote protein turnover in the cytoplasm and nucleus, while mitochondrial proteins can be eliminated by specific proteases (23,29,30). In addition to the changes measured with respect to protein translation, our proteomics data showed that two major mitochondrial proteases (i.e. Lon and Clp) were upregulated upon ATM depletion (Table 2). The most striking phenotype, however, was displayed by the proteasome. In fact, analysis of our data highlighted an increased amount of virtually every subunit constituting the 26S proteasome, specifically in the nuclear compartment of ATM-depleted cells (Figure 3A). We validated this phenotype using different ATM-targeting siRNAs and independent approaches. Western blot analyses confirmed upregulation of regulatory (i.e. PSMD13), structural (i.e. PSMA5) as well as catalytic (i.e. PSMB5) subunits in the nuclear compartment of cells depleted of ATM (Figure 3B). Additionally, PSMD13 accumulation in the nucleus was confirmed using confocal microscopy (Figure 3C) and high-throughput immunofluorescence (Supplementary Figure S3A). This phenotype was completely reversed after ATM restoration (Supplementary Figure S3B), although it persisted much longer than ROS and protein carbonylation upon ATM depletion (Figure 1C and Supplementary Figure S2A). Consistent with our data highlighting the presence of oxidised proteins in patient-derived cells (Supplementary Figure S2B), nuclear accumulation of PSMD13 was also observed in cells obtained from an A-T patient (Supplementary Figure S3C). Nuclear accumulation of the proteasome subunit did not occur in response to ATM inhibition (Supplementary Figure S3D). We concluded that an upregulation in 26S proteasome components was consistent with the accumulation of oxidised proteins observed mainly in the nuclear compartment of ATM-depleted fibroblasts (Figure 1E) and prompted us to investigate this phenotype further.

Increased nuclear proteasomal activity may affect the levels of proteins resident in the nuclear compartment. For example, proteasome activity has been shown to play an important role in histone turnover, particularly upon oxidative damage (31,32). Analysis of our proteomic data highlighted a substantial reduction in the levels of all the histone species (Figure 3D). This observation was validated by Western blot, analysing histone content in acid-extracted protein fractions obtained from ATM-depleted cells (Figure 3E). Furthermore, acid-extracted protein fractions showed higher levels of carbonylation upon ATM depletion (Supplementary Figure S3E). This suggests that increased proteasome levels in the nuclear compartment may contribute to a faster turnover of oxidized histones in cells lacking ATM. Accumulation of the proteasome subunit PSMD13 in the nuclear compartment in cells treated with ROS-generating agents (i.e. paraquat, H_2_O_2_ – Supplementary Figure S3F) further supports this hypothesis, suggesting that the nuclear proteasome might indeed contribute to the elimination of oxidized proteins.

### Proteolytic activity is critical for survival of ATM-depleted cells

**Figure 4.**
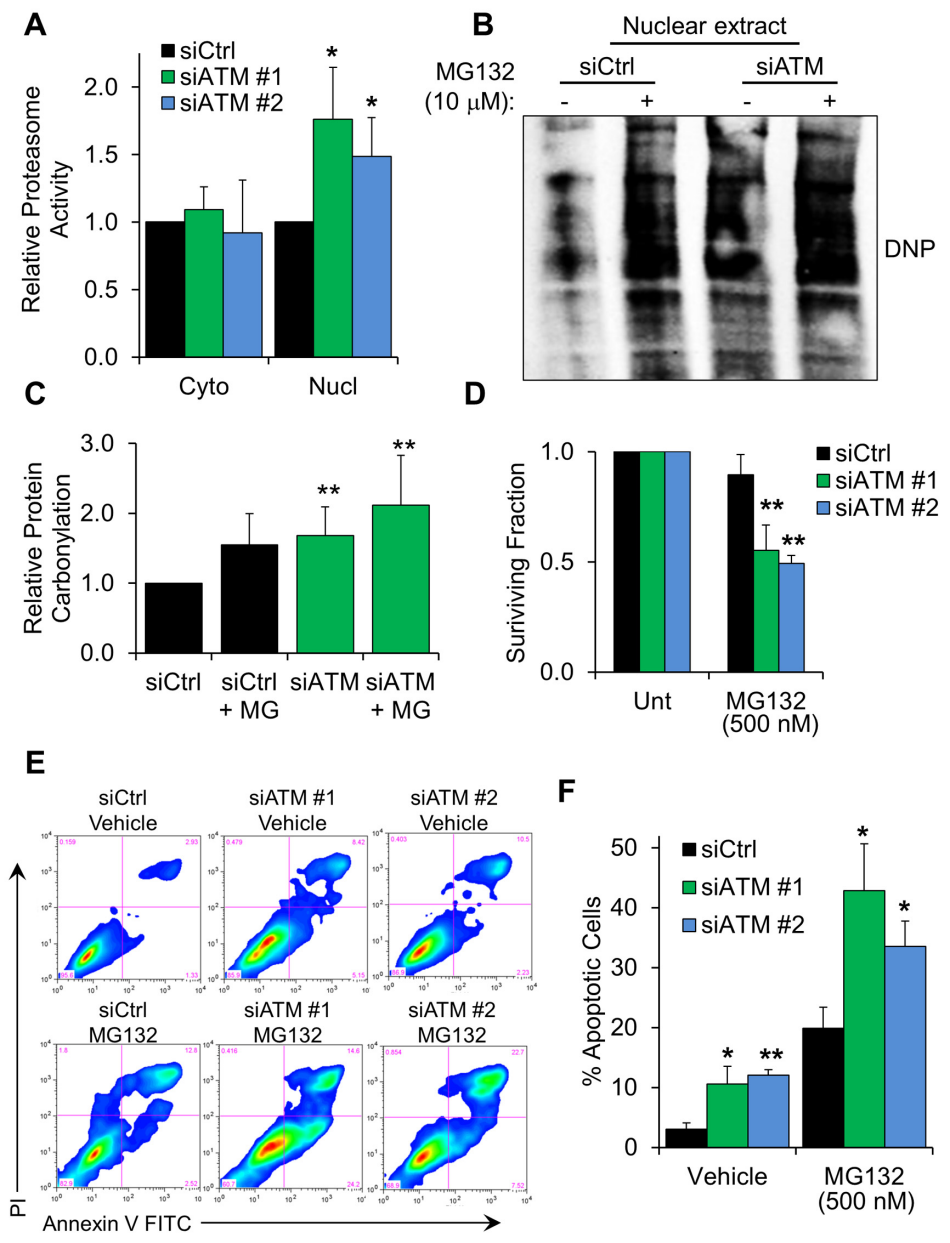
Proteasome activity is required for survival of ATM-depleted fibroblasts. (**A**) Chymotrypsin-like proteasomal activity measured in either cytoplasmic or nuclear cell extracts from TIG1 cells. Protein extraction was carried out 72 hours after transfection with the indicated siRNA (*N* = 3). (**B**) Representative Western blot showing accumulation of carbonylated proteins in the nuclear compartment of fibroblasts depleted of ATM, upon inhibition of proteasomal activity. MG132 treatment (10 μM, 6 h) was carried out 72 h after siRNA transfection. Equal amounts of cell extract were loaded in each lane. (**C**) Densitometric quantification of protein carbonylation in the experiment showed in panel B (*N* = 8). (**D**) Viability assay showing fibroblast sensitivity to proteasome inhibition. TIG1 fibroblasts were transfected with the indicated siRNA; 48 hours after transfection cells were incubated with MG132 (500 nM) for further 24 h. Cell viability was measured using a Trypan Blue exclusion assay (*N* = 4). (**E**) Representative FACS profiles of cells stained with Annexin V and propidium iodide (PI). Cells were treated as in panel D before staining. (**F**) Percentage of apoptotic cells obtained from FACS analysis of cells treated as in panel E (*N* = 3). Results are expressed as mean ± SD from the indicated number (*N*) of independent experiments: *P < 0.05; **P < 0.01.

In order to study the role of the nuclear proteasome in ATM-deficient cells, we measured the proteasome-specific chymotrypsin-like activity in fractionated cell extracts obtained from fibroblasts depleted of ATM (Figure 4A). Higher protease activity, found specifically in the nuclear compartment, was entirely consistent with our model. This also demonstrated that the fraction of the proteasome accumulating in the nucleus of ATM-deficient cells was fully active. We postulated that proteasomal activity in the nucleus could be essential for the survival of fibroblasts lacking ATM. Proteasome inhibition experiments showed that carbonylated proteins accumulate significantly in ATM-depleted nuclear cell extracts when proteasomal activity is impaired (Figure 4B and C). Consistent with this idea, ATM-depleted fibroblasts displayed hypersensitivity to proteasome inhibitors such as MG132 (Figure 4D) and bortezomib (Supplementary Figure S4A); this phenotype was also observed in cells derived from an A-T patient (Supplementary Figure S4B). Furthermore, flow cytometry analyses highlighted an increased number of apoptotic cells under basal conditions, after depletion of ATM (Figure 4E and F). As expected, treatment of these cells with MG132 triggered a strong apoptotic response, 1.5–2 higher as compared with control cells (Figure 4E and F), indicating that proteasomal activity is extremely important for cell survival when ATM function is impaired.

These data show that depletion of ATM leads to accumulation of a fully functional 26S proteasome in the nucleus. This mechanism is likely in place to promote the turnover of oxidized nuclear proteins. In line with this idea, proteasome activity was essential to promote cell survival upon ATM depletion.

### Modulation of proteostasis in ATM-depleted cells leads to reduced BER capacity and spontaneous accumulation of DNA strand breaks

**Figure 5.**
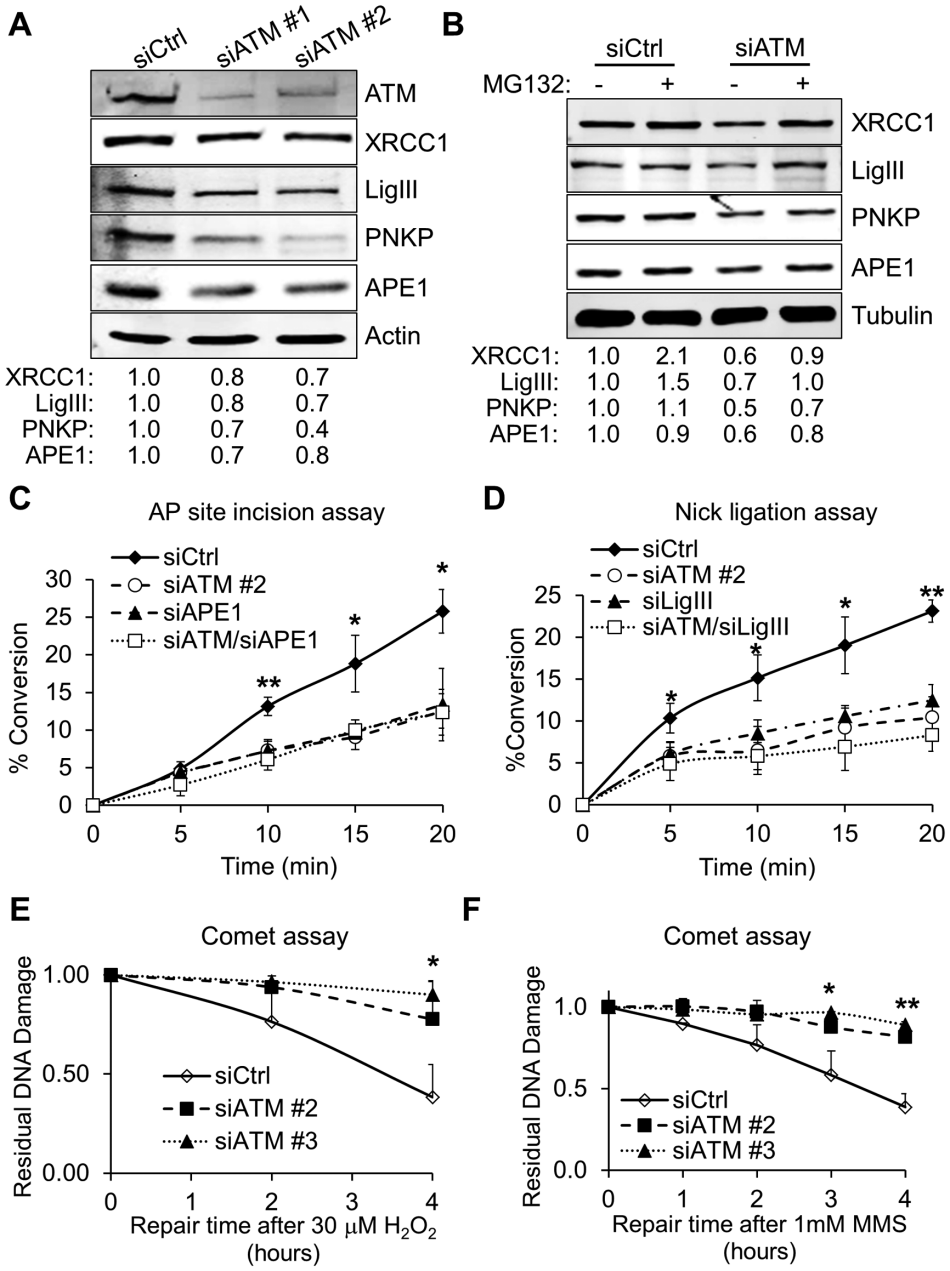
Reduced BER capacity in ATM-depleted fibroblasts. (**A**) Representative Western blot comparing BER protein levels in whole cell extracts from TIG1 cells. Protein extraction was carried out 72 h after transfection with the indicated siRNA. Fold change in protein expression relative to the control sample (siCtrl) is indicated at the bottom of the blot. Actin was used as loading control (*N* = 4). (**B**) Representative Western blot comparing BER protein levels in whole cell extracts from TIG1 cells depleted of ATM in the presence of proteasome inhibitor. MG132 treatment (10 μM, 6 h) was carried out before cell harvesting, 72 h after transfection with the indicated siRNA. Fold change in protein expression relative to the control sample is indicated at the bottom of the blot. Tubulin was used as loading control (*N* = 3). (**C**) AP site incision assay showing impaired endonuclease activity at abasic sites in cells treated with the indicated siRNA. The assay was carried out as described in ‘Materials and Methods’ using whole cell extracts obtained from cells treated as indicated (*N* = 3). (**D**) Nick ligation assay showing impaired SSB ligation activity in ATM-depleted cells. The assay was carried out as described in ‘Materials and Methods’ using nuclear cell extracts obtained from cells treated with the indicated siRNA (*N* = 3). (**E**) Alkaline comet assay showing slower DNA repair kinetics for ATM-depleted cells. TIG1 fibroblasts were transfected with the indicated siRNA; 72 h after transfection cells were treated with H_2_O_2_ (30 μM, 10 min) and allowed to repair for the indicated amount of time. DNA repair is expressed as fraction of residual DNA damage after H_2_O_2_ treatment (*N* = 3). (**F**) Alkaline comet assay showing slower DNA repair kinetics in ATM-depleted cells. TIG1 fibroblasts were treated as in panel E using MMS (1 mM, 10 min) and allowed to repair for the indicated amount of time. DNA repair is expressed as fraction of residual DNA damage after MMS treatment (*N* = 3). Results are expressed as mean ± SD from the indicated number (*N*) of independent experiments: *P < 0.05; **P < 0.01.

In addition to proteins, DNA is particularly sensitive to oxidative stress; ATM activation in response to oxidative DNA damage and SSBs has been extensively documented (6,33,34). Accordingly, a number of studies have suggested the existence of cross-talk between ATM and BER, the major DNA repair system responsible for clearance of SSBs and oxidative DNA lesions (6,34). Given that fibroblasts lacking ATM displayed a very high protein turnover, we asked whether BER capacity could be affected by ATM depletion. Surprisingly, a number of essential BER components, including DNA ligase IIIα (LigIII), polynucleotide kinase 3′-phosphatase (PNKP), X-ray repair cross complementing 1 (XRCC1) and apurinic/apyrimidinic endonuclease 1 (APE1) were downregulated in response to ATM depletion (Figure 5A), as well as in fibroblasts derived from an A-T patient (Supplementary Figure S5A). However, consistent with the absence of oxidative stress (Figure 1), or proteostatic rearrangements (Figures 2, 3 and 4), BER downregulation was not observed when ATM’s kinase activity was inhibited in normal fibroblasts (Supplementary Figure S5B). Importantly, BER downregulation was rescued by proteasome inhibition (Figure 5B), suggesting that increased proteasome activity was responsible for the BER suppression in ATM-depleted fibroblasts. In line with this idea, downregulation of BER components persisted for several days following ATM depletion (Supplementary Figure S5C), matching the kinetics of recovery observed for the proteasome subunit PSMD13 (Supplementary Figure S3B).

Downregulation of core BER components is likely to impact BER-mediated repair. In order to test this, we exploited *in vitro* DNA repair assays where lesion-containing oligonucleotides were incubated with ATM-depleted cell extracts to interrogate different steps of the BER pathway. Abasic (AP) site incision assays, mainly evaluating APE1′s AP-endonuclease activity, revealed that ATM-depleted cell extracts indeed had reduced AP-endonuclease capacity (Figure 5C). This reduction in DNA repair capacity was efficiently mimicked by partial depletion of APE1, and was not further exacerbated by simultaneous suppression of APE1 and ATM (Figure 5C and Supplementary Figure S5D). Additionally, the ability of ATM-depleted cells to ligate a nicked DNA substrate was also significantly affected (Figure 5D and Supplementary Figure S5E). Again, reduction in DNA ligation efficiency did not show additional impairment when ATM and LigIII were depleted simultaneously. We concluded that the deficiency in DNA repair observed was entirely consistent with the lower levels of APE1, XRCC1 and LigIII measured upon ATM knock-down (Figure 5A). In order to further substantiate the condition of BER impairment in ATM-depleted cells, we measured DNA repair in cells treated with H_2_O_2_, or the alkylating agent methyl methanesulfonate (MMS). DNA damage was assessed over a recovery time-course upon cell treatment with the relevant genotoxin, by means of alkaline comet assays. Consistent with a profound BER impairment, ATM-depleted cells were significantly slower than control cells in repairing damage induced by both H_2_O_2_ (Figure 5E) and MMS (Figure 5F). Altogether, these data demonstrate that BER is strongly impaired in fibroblasts lacking ATM and that this phenotype is likely to be a consequence of modified proteostasis in these cells. In line with these observations, cells lacking ATM have been reported to display hypersensitivity to a wide range of BER-eliciting genotoxins including oxidative (35–37) and alkylating (38) agents.

**Figure 6.**
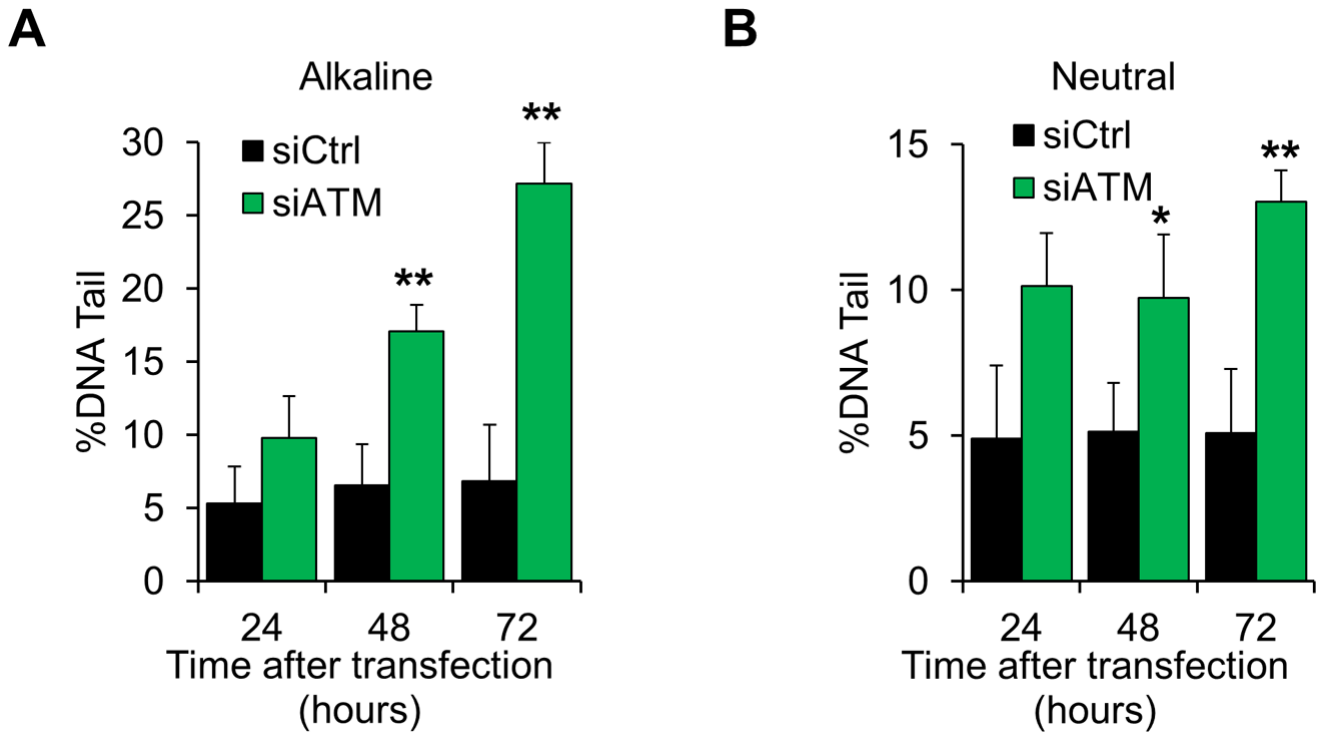
Spontaneous accumulation of DNA strand breaks in ATM-deficient cells. (**A**) Alkaline comet assay on TIG1 cells depleted of ATM for the indicated amount of time. The amount of DNA damage, expressed as percentage of DNA in the comet tails, increases in a time-dependent manner (*N* = 3). (**B**) Neutral comet assay on TIG1 cells treated as in panel A. The amount of DNA damage, expressed as percentage of DNA in the comet tails, increases in a time-dependent manner (*N* = 3). Results are expressed as mean ± SD from the indicated number (*N*) of independent experiments: *P < 0.05; **P < 0.01.

Exhaustion of DNA repair, BER in particular, has been linked to genomic instability and accumulation of endogenously generated DNA strand-breaks (6,13,15). We hypothesized that reduced DNA repair capacity could lead to spontaneous accumulation of unrepaired DNA lesions in ATM-depleted fibroblasts. In line with this hypothesis, fibroblasts treated with an ATM-targeting siRNA showed a progressive accumulation of spontaneous DNA damage (Figure 6A and Supplementary Figure S6A), which was comparable to the amount of damage generated by 30 μM H_2_O_2_ (Supplementary Figure S6B) and entirely consistent with the amount of ROS measured in ATM-depleted cells (compare with Figure 1C). As accumulation of DNA damage was readily detected using both alkaline and neural comet assays (Figure 6B and Supplementary Figure S6C), we concluded that ATM depletion leads to the accumulation of spontaneous DNA damage in normal fibroblasts, which consist of both SSBs and DNA double-strand breaks (DSBs).

Taken together, these data show that lack of ATM leads to increased protein turnover, which—in turn—results in a downregulation of BER components. This evidence suggests that decline in DNA repair capacity is one of the early mechanisms driving accumulation of genomic instability in ATM-depleted fibroblasts.

## DISCUSSION

Genomic instability in cells lacking functional ATM is a well-established feature (1). Stable knock-down and A-T patient-derived cell lines have been widely used to study the consequences of a lack of functional ATM. However, the presence of extensive genomic rearrangements in these cell models can be source of bias when interpreting experimental data. For this reason, our study was carried out using a short-term ATM depletion model, where genomic instability and adaptation phenomena are not already established. Our experimental setup allowed us to assess proteomics variations occurring very early on after ATM depletion and this highlighted initial changes that may have been overlooked by previous analyses.

The first impression emerging from our analysis indicates that ATM depletion very rapidly leads to the generation of oxidative stress. Our study confirms previous observations from mouse models (8,20) and human patients (9), highlighting the importance of oxidative stress in A-T. While our study does not pinpoint the molecular mechanism leading to ROS accumulation upon ATM depletion, earlier research has shown that ATM itself coordinates cellular redox homeostasis through a variety of mechanisms including direct ROS sensing, production of antioxidant molecules and oxidative stress signalling (reviewed in (39)). Therefore we suggest that, at the steady state, ATM is crucial to keep in check endogenous ROS levels. The lack of such a pivotal molecule is likely sufficient to disrupt redox homeostasis very quickly. We demonstrate here for the first time that generation of ROS in cells depleted of ATM leads to the accumulation of oxidised proteins. Control and prevention of protein oxidation are generally achieved through modulation of proteostasis. To our surprise, cells lacking ATM show a very remarkable plasticity, putting in place different mechanisms specifically aiming to limit protein damage. Our data demonstrate, for example, that ATM-depleted fibroblasts reduce the rate of general protein synthesis and upregulate specific aaRSs. These mechanisms are likely in place to minimize the synthesis of polypeptides that may incorporate oxidized amino acids. An even more striking phenotype, however, is the selective redirection of cellular proteolysis to the nuclear compartment. Previous studies have hinted at a potential role for ATM in regulating the ubiquitin proteasome system. For example, ATM has been suggested to promote proteasome recruitment to the nucleus through phosphorylation of the nuclear proteasome activator PSME3 (PA28γ) (40). In our case, though, it is entirely plausible that the observed widespread nuclear accumulation of proteasome particles is actually the consequence of a broader transcriptional activation of proteasome genes in response to persistent oxidative stress (41). Another study highlighted a profound deregulation in the ubiquitin–proteasome system (UPS) in A-T cells (42); in this case, overexpression of the ubiquitin-like modifier ISG15 was suggested to affect proteostasis (42). Consistent with this idea, our proteomics analysis also detected a higher amount of ISG15 in ATM-depleted fibroblasts (Table 2), confirming a potential role for the molecule in the pathogenesis of A-T. Incidentally, ISG15 has been demonstrated to downregulate general protein translation (18). This is also entirely consistent with the observations reported herein, and points to a cross-talk between proteolysis and protein synthesis. Furthermore, our data highlight the strong relevance of proteolytic activity for the survival of ATM-depleted fibroblasts, as these cells show hypersensitivity to proteasome inhibitors.

This study demonstrates that persistent oxidative stress in ATM-depleted cells is essentially well buffered by adjustments in proteostasis. This potentially represents an effective solution to promote cell survival in the short term. The other side of the coin is that a broad rearrangement in proteostasis will inevitably lead to unwanted deregulation in the long term. This is exemplified by DNA repair capacity, which is severely impaired as a consequence of the increased proteolytic activity. BER in particular, has been poorly studied in A-T cells. To the best of our knowledge, a single study investigated BER in A-T lymphoblasts transformed with the Epstein-Barr virus, concluding that these cells bear normal BER capacity (43). However, we have recently shown that viral transformation alters BER coordination (13). Therefore, it is conceivable that transformed cells will not respond to ATM depletion in the same way as normal cells. Consistent with this idea, our study shows that loss of ATM indirectly affects BER capacity in normal fibroblasts. This, in turn, could be one of the factors contributing to progressive genomic instability, which we have measured here in the form of DNA strand breaks. Interestingly, the general response observed upon ATM depletion in diploid fibroblasts was also recapitulated in fibroblasts obtained from A-T patients, suggesting that most of the phenotypes we observe here bear clinical relevance. On the other hand, it is important to consider that fibroblasts are not necessarily representative of all cell types; therefore, follow up studies using cell types more relevant to the A-T pathology (e.g. neurons, or immune cells) will be needed in order to reach definitive conclusions. Furthermore, while our data suggest that cellular adaptations to a lack of ATM can potentially be reversed by re-expression of the protein, it would be of great interest to understand whether such a reversion could be achieved in cells that have experienced a prolonged absence of ATM.

The importance of BER for cell survival is well established; in particular, a number of studies have shown that BER capacity is implicated in the maintenance of neurological function (44–46). Crucially, a very recent publication linked together XRCC1 mutation and occurrence of cerebellar ataxia (46), which is a hallmark of A-T (1). In this context, our data could provide further insight into the progressive neurodegeneration observed in A-T patients, as accumulation of endogenously generated DNA damage, together with impairment of DNA repair capacity, could promote neurodegeneration in individuals lacking ATM. At the same time, BER function is crucial to promote genomic stability, as endogenous DNA damage occurs with very high frequency (11,47). Therefore, impaired DNA repair capacity is expected to increase the rate of spontaneous mutagenesis and cancer. As a consequence, the lower BER capacity observed in cells lacking ATM could be one of the contributing factors leading to susceptibility to cancer in A-T individuals. It is important to highlight that while our study focussed on the BER pathway, our data do not exclude the possibility that other DNA repair pathways are also negatively affected by a lack of ATM. In fact, widespread upregulation of nuclear proteolysis is likely to have broader impact on DNA repair in these cells.

Interestingly, the phenotypes observed in the absence of ATM did not occur when ATM’s kinase activity was inhibited. This suggests that some yet unknown function of ATM that is not affected by kinase inhibitors is important for cellular homeostasis. In fact, the vast majority of mutations in the *ATM* gene have been shown to lead to the expression of a truncated protein in A-T patients (48), highlighting how the absence of full-length ATM, rather than just its kinase activity, is the most common molecular basis of A-T.

In conclusion, we report here a comprehensive analysis of the adaptation of normal human fibroblasts to a lack of ATM. This study highlights novel characteristics of ATM-depleted cells, emphasising the role of proteostasis in promoting cell survival in the absence of functional ATM. Furthermore, we reveal how the mechanisms in place to counteract protein damage indirectly impact DNA repair capacity, leading to accumulation of endogenous DNA damage and genomic instability. These findings shed light on the emergence of A-T features including neurodegeneration and predisposition to cancer.

## Supplementary Material

Supplementary Data
