# Supplementary material for: Modulation of proteostasis counteracts oxidative stress and affects DNA base excision repair capacity in ATM-deficient cells
Source: Nucleic Acids Res. 2017 Jul 24;45(17):10042–55. doi: 10.1093/nar/gkx635 (PMC5622344; doi:10.1093/nar/gkx635)
Supplement: Supplementary Data [file gkx635_supp.docx]

**Modulation of proteostasis counteracts oxidative stress and affects DNA base excision repair capacity in ATM-deficient cells**

MATTIA POLETTO, DI YANG, SALLY C FLETCHER, IOLANDA VENDRELL, ROMAN FISCHER, ARNAUD J LEGRAND, GRIGORY L DIANOV

**SUPPLEMENTARY DATA**

**Supplementary Materials and Methods**

*siRNA sequences used in this study*

| **Target** | **Sequence (5**' **to 3')** |
| --- | --- |
| APE1 | AAUGACAAAGAGGCAGCAGG |
| ATM #1 | AACAUACUACUCAAAGACAUU |
| ATM #2 | UGGUGCUAUUUACGGAGCU |
| ATM #3 | GCGCCUGAUUCGAGAUCCU |
| LigIII | AACUGCAACCCAGAUGAUAUG |

*Primers used in this study*

| **Target** | **Sequence (5**' **to 3')** |
| --- | --- |
| B2M | For: ATGTCTCGCTCCGTGGCCTTA |
|  | Rev: ATCTTGGGCTGTGACAAAGTC |
| CBR1 | For: TTTTAAGGGCTCTGACGCTC |
|  | Rev: TTTGGTACCCGAGATGTGTG |
| EGFP | For: ACGTAAACGGCCACAAGTTC |
|  | Rev: AAGTCGTGCTGCTTCATGTG |
| GAPDH | For: AGCCACATCGCTCAGACAC |
|  | Rev: GCCCAATACGACCAAATCC |
| GLRX | For: CACTGCATCCGCCTATACAA |
|  | Rev: CAGCCACCAACCACACTAAC |
| GLRX5 | For: TCACAGCCCCCTACAAACTC |
|  | Rev: CCTACAACGTGCTGGACGA |
| PRDX1 | For: GGGCACACAAAGGTGAAGTC |
|  | Rev: GCTGTTATGCCAGATGGTCAG |
| PRDX6 | For: CAAGCTCCCGATTCCTATCA |
|  | Rev: GTTGAGGACCATCTTGCCTG |
| TXNRD1 | For: TCAGGGCCGTTCATTTTTAG |
|  | Rev: GATCTGCCCGTTGTGTTTG |

*Antibodies used in this study*

| **Target** | **Antibody** |
| --- | --- |
| Actin | ab6276 – Abcam |
| APE1 | NB100-101 – Novus Biologicals |
| ATM | A1106 – Sigma |
| ATM (Ser 1981 phosphorylated) | Ab81292 – Abcam |
| CARS | A302-409A-T – Bethyl Laboratories |
| CBR1 | A304-773A-T – Bethyl Laboratories |
| LigIII | Made in house |
| H1 | sc-56695 – Santa Cruz Biotechnology |
| H3 | ab201456 – Abcam |
| Hsp90 | ab34909 – Abcam |
| Ku80 | ab3715 – Abcam |
| Lamin A/C | ab108922 – Abcam |
| PNKP | Kind gift from Prof Michael Weinfeld (University of Alberta) |
| PSMA5 | 2457S – Cell Signaling Technology |
| PSMD13 | 15261-1-AP - Proteintech |
| Tubulin | T6199 – Sigma |
| TXNRD1 | A304-791A-T – Bethyl Laboratories |
| XRCC1 | MS-1393-P0 – Thermo Scientific |

**Supplementary Figures and Figure Legends**


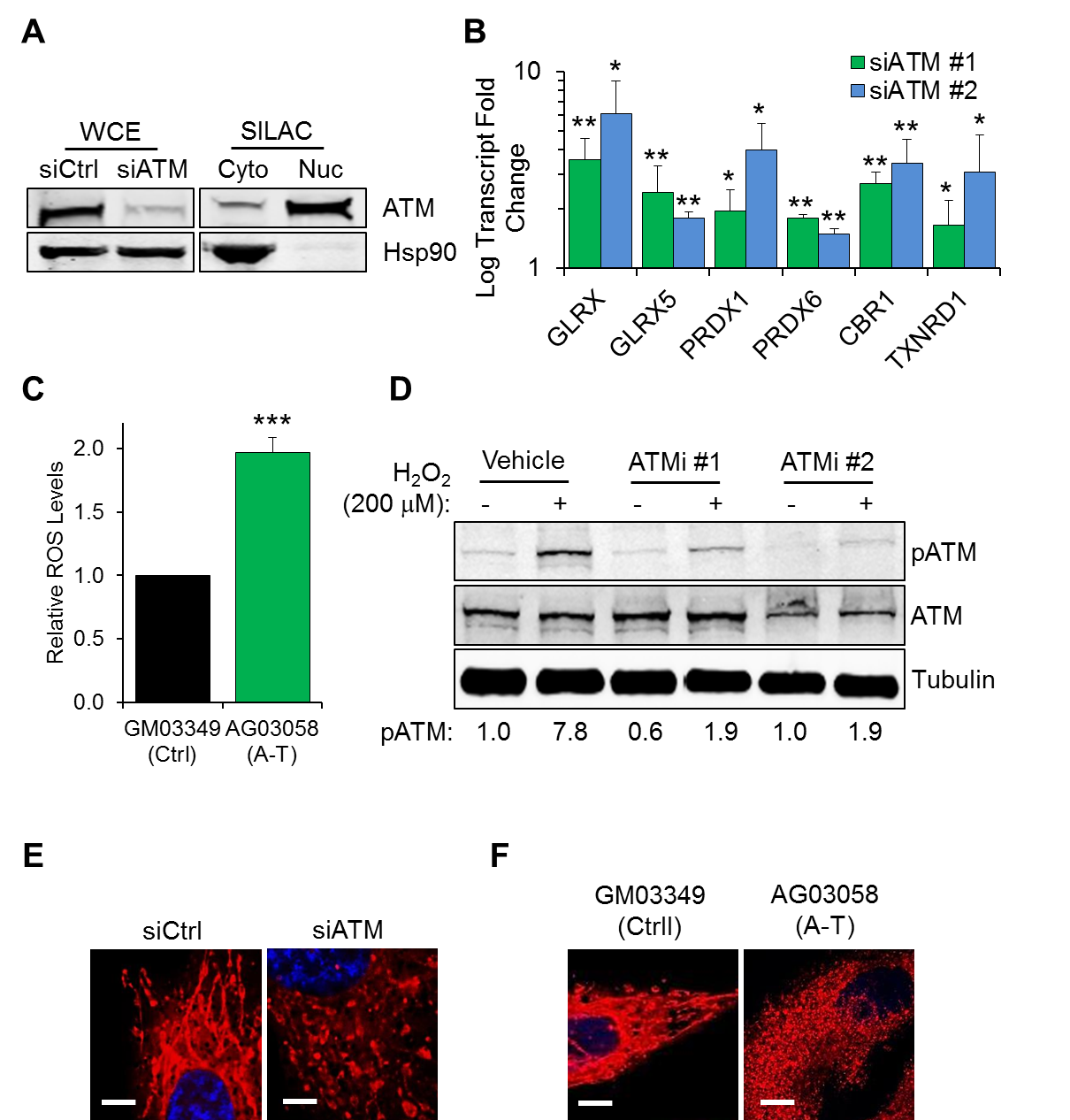


**Supplementary Figure S1 – Fibroblasts lacking ATM display oxidative stress and mitochondrial dysfunction.**

**(A)** Representative Western blot analysis on samples subjected to SILAC proteomics. Whole cell extracts (WCE) treated with a control siRNA (siCtrl) or with an ATM-targeting siRNA (siATM) are shown on the left-hand side. Hsp90 was used as loading control. siCtrl- and siATM-treated samples were pooled and subjected to fractionation into cytoplasmic and nuclear compartments, as illustrated on the right-hand side. **(B)** qPCR validation of the upregulation measured by proteomics on a subset of antioxidant genes. The expression level of selected antioxidant genes was measured 72 hours after transfection with the indicated ATM-targeting siRNA. The histogram reports the Log_(10)_ fold change normalised to the control siRNA. **(C)** Quantification of ROS content using flow-cytometry in normal and A-T fibroblasts (N=4). **(D)** Representative Western blot analysis validating the activity of the ATM inhibitors used in this study. Cells were treated with either vehicle (DMSO), Ku-55933 (ATMi #1, 10 μM), or Ku-60019 (ATMi #2, 10 μM) which were fed fresh every 24 hours for a total of 72 hours. H_2_O_2_ (200 μM, 30 minutes) was used to induce ATM auto-phosphorylation at serine 1981 (pATM), which was monitored as a marker for ATM kinase activity. Densitometric quantification is reported at the bottom and expressed as pATM/ATM/Tubulin ratio. **(E)** Representative micrographs showing fragmentation of the mitochondrial network in cells depleted for ATM. Mitochondria (red) were stained with MitoTracker^®^ Red; nuclei (blue) were stained with Hoechst. Scale bars 5 μm. **(F)** Representative micrographs showing fragmentation of the mitochondrial network in A-T cells. Cells were stained as in panel E. Scale bars 5 μm. Results are expressed as mean from the indicated number (N) of independent experiments * p<0.05; ** p<0.01; *** p<0.001.

**
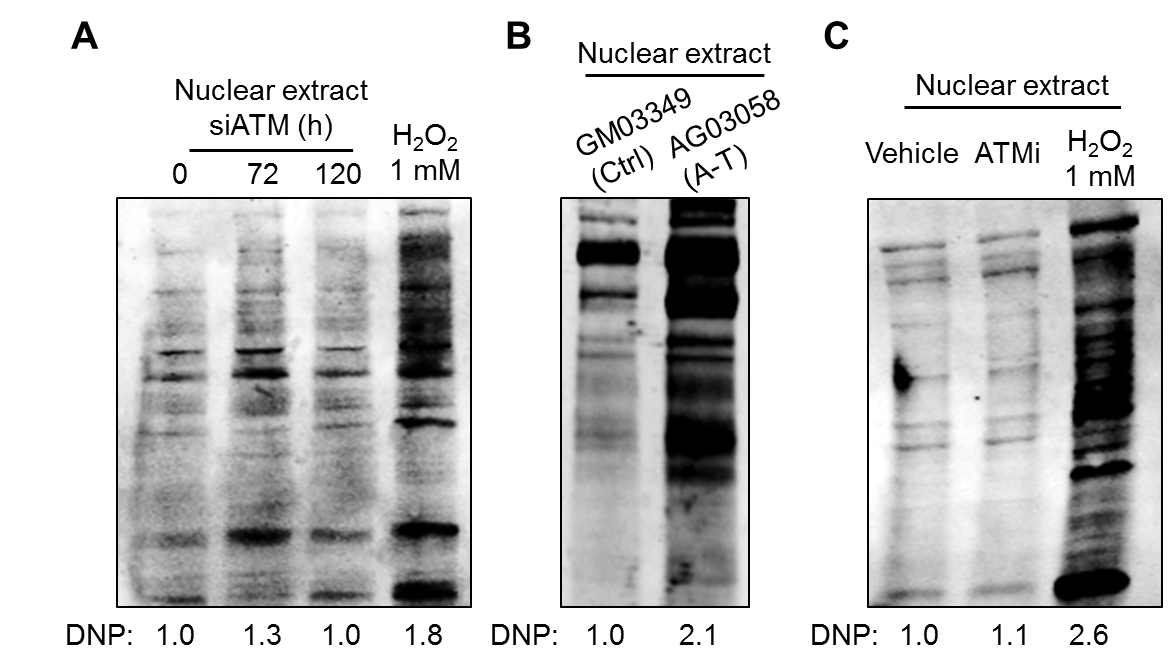
**

**Supplementary Figure S2 – Cells lacking ATM show accumulation of carbonylated proteins.**

**(A)** Quantification of protein carbonylation in nuclear extracts obtained from fibroblasts depleted of ATM for the indicated time. H_2_O_2_ (1 mM, 30 minutes) was used as a positive control for induction of protein carbonylation (N=2). **(B)** Quantification of protein carbonylation in nuclear extracts obtained from either normal or A-T fibroblasts (N=3). **(C)** Quantification of protein carbonylation in nuclear extracts obtained from fibroblasts treated with the ATM inhibitor Ku-55933 as in Supplementary Figure S1. H_2_O_2_ (1 mM, 30 minutes) was used as a positive control for induction of protein carbonylation (N=2). Densitometric quantification is reported at the bottom of each gel; equal amounts of cell extract were loaded in each lane. Results are expressed as mean from the indicated number (N) of independent experiments.


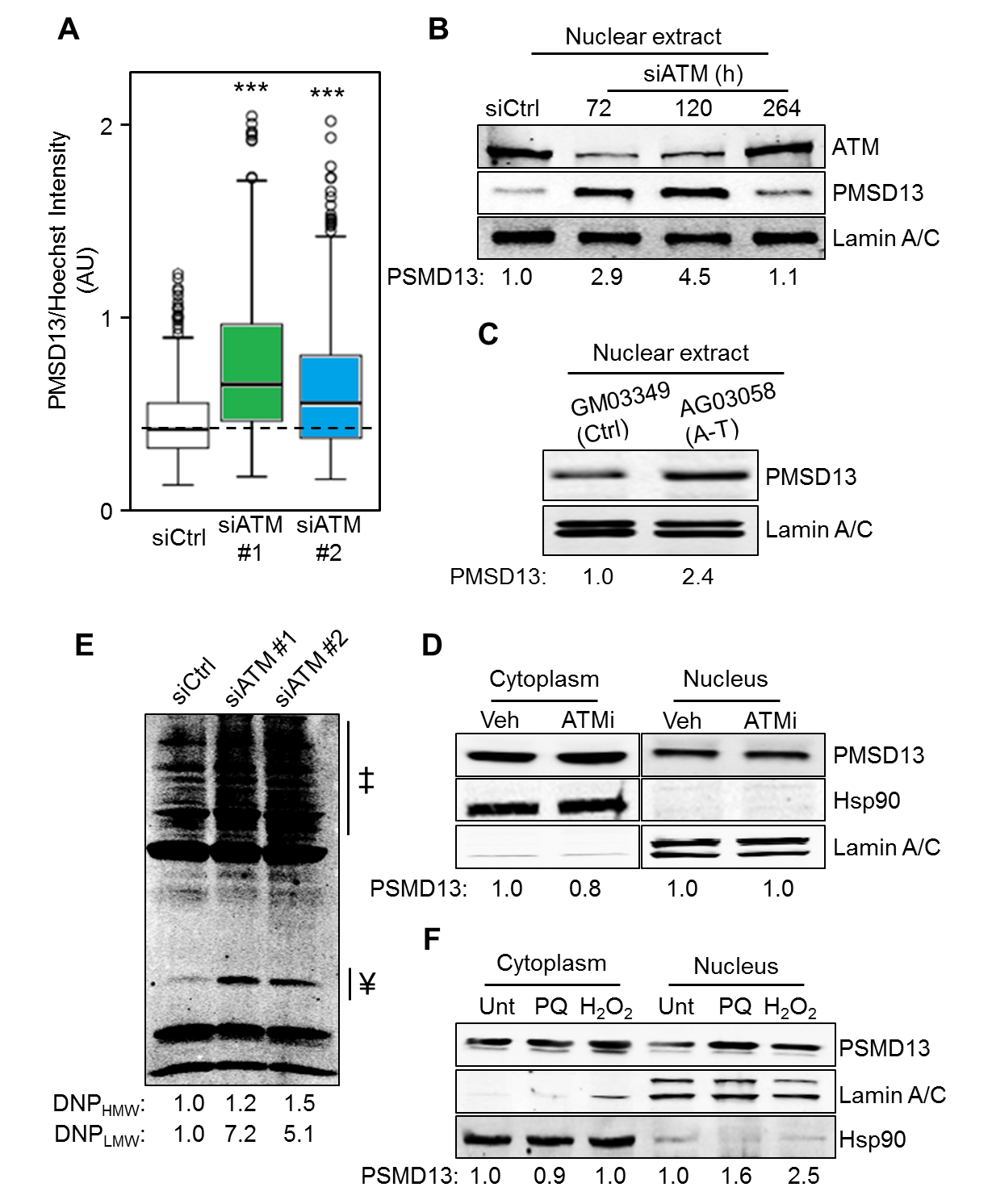


**Supplementary Figure S3 – Accumulation of the proteasome in the nuclear compartment of ATM-depleted cells.**

**(A)** Representative quantification of the extent of nuclear localisation of the proteasome subunit PMSD13 upon ATM depletion. Nuclear PSMD13 fluorescence intensity was measured by high throughput immuno-fluorescence and normalised to the fluorescence intensity of Hoechst. Median nuclear PSMD13 fluorescence was measured in at least 500 cells *** p<0.001 (Kruskal-Wallis test). **(B)** Representative Western blot analysis assessing nuclear PSMD13 content in cells treated with an ATM-targeting siRNA for the indicated amount of time. PSMD13 returns to basal levels between 6 and 11 days after ATM depletion. Densitometric quantification is reported at the bottom; Lamin A/C was used as a loading control (N=2). **(C)** Representative Western blot analysis comparing nuclear PSMD13 content in normal and A-T fibroblasts. Densitometric quantification is reported at the bottom; Lamin A/C was used as a loading control (N=3). **(D)** Representative Western blot analysis assessing nuclear PSMD13 content in cells treated with the ATM inhibitor Ku-55933 as in Supplementary Figure S1. Densitometric quantification is reported at the bottom; Hsp90 and Lamin A/C were used as loading controls for the cytoplasmic and nuclear compartment, respectively (N=3). **(E)** Representative Western blot analysis on histone-enriched fractions obtained from cells treated with the indicated siRNA. Carbonylation levels were assessed by loading equal amounts of acid-extracted protein fractions and probing with an anti-DNP antibody, as described in Figure 1. Densitometric quantification of the high molecular weight (‡ DNP_HMW_) and of the low molecular weight (¥ DNP_LMW_) signals is reported at the bottom (N=2). **(F)** Representative Western blot analysis showing increased amount of PSMD13 in the nuclear compartment of fibroblasts treated with either paraquat (PQ – 150 μM) or H_2_O_2_ (125 μM). Cells were treated for 72 hours; drugs were supplied fresh every 24 hours. Hsp90 and Lamin A/C were used as loading controls for the cytoplasmic and nuclear compartment, respectively. Unt: untreated (N=3). Results are expressed as mean calculated from the indicated number (N) of independent experiments.


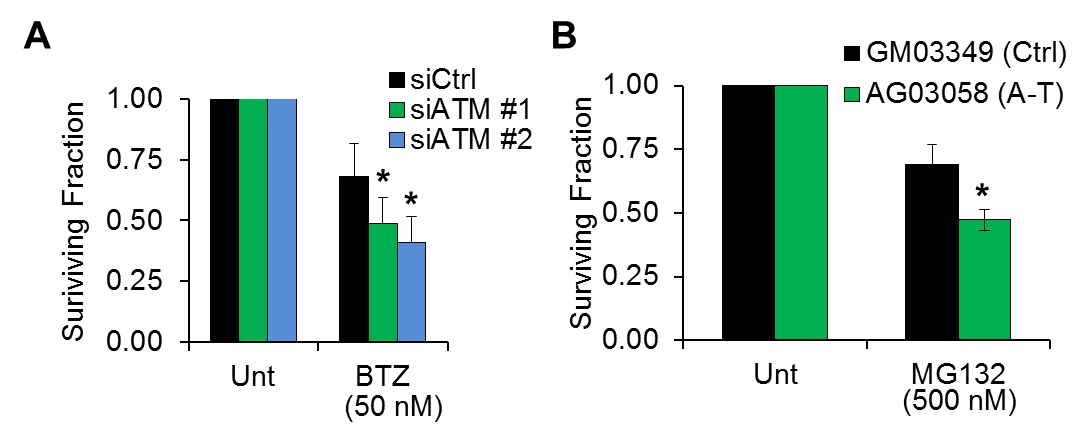


**Supplementary Figure S4 – Proteolytic activity is required for survival of fibroblasts lacking ATM.**

**(A)** Viability assay showing fibroblast sensitivity to proteasome inhibition. TIG1 fibroblasts were transfected with the indicated siRNA; 48 hours later cells were incubated with bortezomib (BTZ – 50 nM) for further 24 hours. Cell viability was measured using a Trypan Blue exclusion assay (N=3). **(B)** Viability assay showing fibroblast sensitivity to proteasome inhibition. Either normal or A-T fibroblasts were incubated with MG132 (500 nM, 24h). Cell viability was measured using a Trypan Blue exclusion assay (N=3). Results are expressed as mean ± SD from the indicated number (N) of independent experiments * p<0.05.


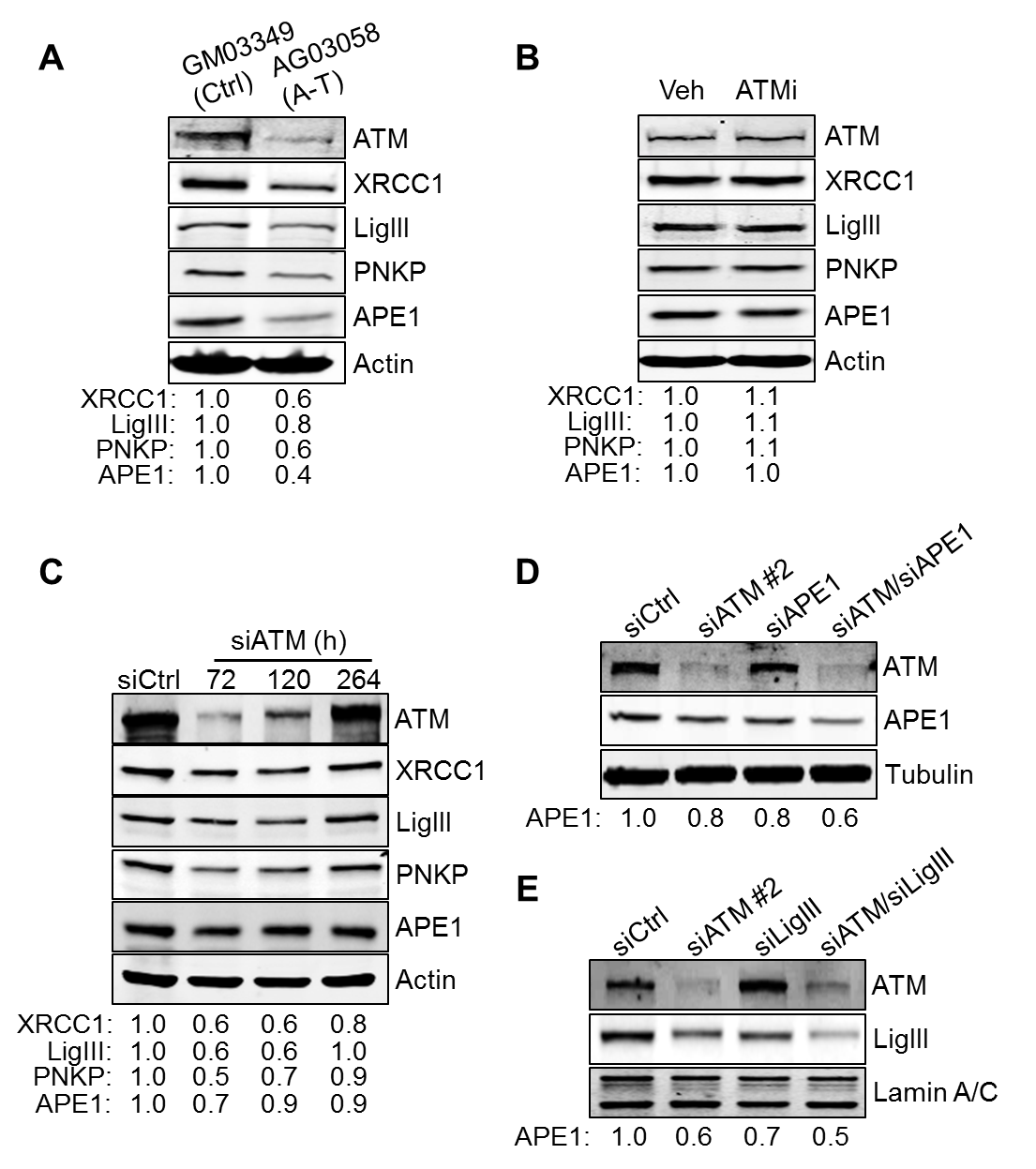


**Supplementary Figure S5 – Reduced BER capacity in ATM-depleted fibroblasts.**

**(A)** Representative Western blot showing reduction in BER components in cells from an A-T patient (N=3). **(B)** Representative Western blot analysis showing unchanged levels of BER components upon cell treatment with an ATM inhibitor (Ku-55933, 10 μM fed fresh every 24 hours for a total of 72 hours) (N=3). **(C)** Representative Western blot analysis assessing the levels of BER proteins in cells treated with an ATM-targeting siRNA for the indicated amount of time. BER components return to basal levels between 6 and 11 days after ATM depletion. **(D)** Representative Western blot analysis showing APE1 and ATM levels in cells treated with the indicated siRNA. To achieve partial APE1 suppression, transfection was carried out as described in “Materials and methods” (N=2). **(E)** Representative Western blot analysis showing LigIII and ATM levels in cells treated with the indicated siRNA. To achieve partial LigIII suppression, transfection was carried out as described in “Materials and methods” (N=2). Densitometric quantification is reported at the bottom of each gel; actin, lamin A/C, or tubulin was used as loading control. Results are expressed as average from the indicated number (N) of independent experiments.


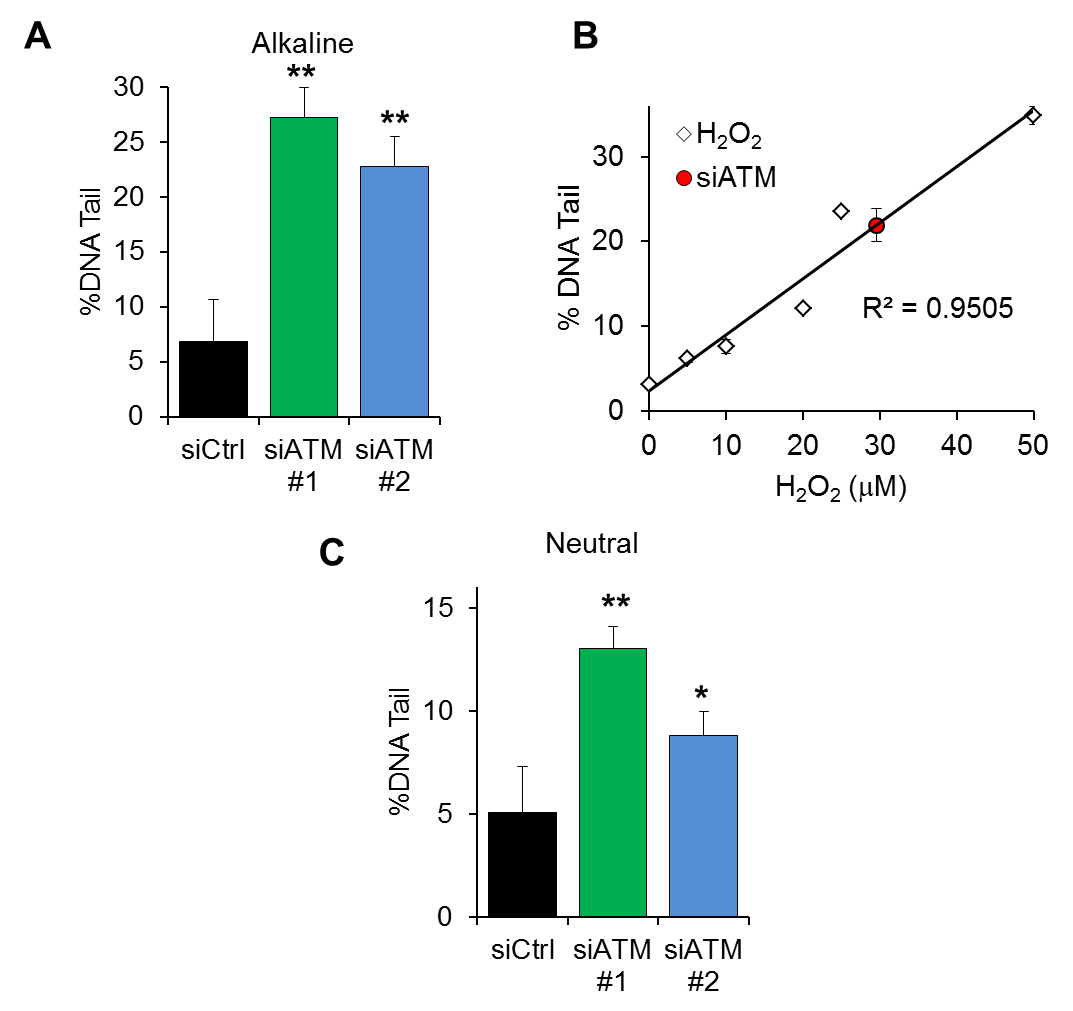


**Supplementary Figure S6 – Spontaneous accumulation of DNA strand breaks in ATM-deficient cells.**

**(A)** Alkaline comet assay on TIG1 cells depleted of ATM using the indicated siRNA. The amount of DNA damage, expressed as a percentage of DNA in the comet tail, was measured 72 hours post-transfection (N=3). **(B)** Alkaline comet assay run as in panel D. A titration curve was generated treating cells with increasing amounts of H_2_O_2_ (5 minutes, on ice) and the load of damage generated by ATM depletion was extrapolated from the trendline. **(C)** Neutral comet assay carried out as in panel D (N=3). Results are expressed as mean ± SD from the indicated number (N) of independent experiments * p<0.05; ** p<0.01.
